# Malaria and Nutritional Status Among Children With Severe Acute Malnutrition in Niger: A Prospective Cohort Study

**DOI:** 10.1093/cid/ciy207

**Published:** 2018-03-07

**Authors:** Catherine E Oldenburg, Philippe J Guerin, Fatou Berthé, Rebecca F Grais, Sheila Isanaka

**Affiliations:** 1 Francis I. Proctor Foundation, University of California, San Francisco; 2 Department of Ophthalmology, University of California, San Francisco; 3 WorldWide Antimalarial Resistance Network, University of Oxford, United Kingdom; 4 Centre for Tropical Medicine and Global Health, Nuffield Department of Medicine, University of Oxford, United Kingdom; 5 Epicentre, Maradi, Niger; 6 Department of Research, Epicentre, Paris, France; 7 Departments of Nutrition and Global Health and Population, Harvard T.H. Chan School of Public Health, Boston, Massachusetts

**Keywords:** severe acute malnutrition, malaria, Niger

## Abstract

**Background:**

The relationship between malaria infection and nutritional status is complex. Previous studies suggest malaria may increase the incidence and severity of malnutrition, while malnutrition may increase the risk of malaria infection. Here, we report bidirectional associations between malaria and nutritional status among children with uncomplicated severe acute malnutrition (SAM).

**Methods:**

This study is a secondary analysis of a randomized, controlled trial for the treatment of uncomplicated SAM in Niger. Children aged 6–59 months were enrolled and followed for 12 weeks. Malaria infection was assessed using an histidine-rich protein 2 (HRP2) rapid diagnostic test at admission and at any follow-up visit with fever. We assessed the association of nutritional status at admission on malaria incidence using Cox proportional hazards regression and malaria infection at admission on nutritional recovery and weight and height gain using linear regression.

**Results:**

Of 2399 children included in the analysis, 1327 (55.3%) were infected with malaria at admission. Malaria incidence was 12.1 cases/100 person-months among those without malaria infection at admission. Nutritional status at admission was not associated with malaria incidence. Children with malaria infection at admission and subsequently treated with an artemisinin-based combination therapy had increased weight gain (0.38 g/kg/day; 95% confidence interval [CI], 0.07 to 0.69) and reduced height gain (−0.002 mm/day; 95% CI, −0.004 to −0.0008).

**Conclusions:**

Malaria infection was common among children treated for uncomplicated SAM. Malaria infection may impair height gain. Proper medical and nutritional management should be ensured to prevent adverse effects of malaria infection.

**Clinical Trials Registration:**

NCT01613547.

## Teaser

We assessed the relationships between nutritional status and malaria infection among children with severe acute malnutrition. Baseline nutritional status was not associated with malaria infection. Malaria infection was associated with increased weight gain and decreased linear growth.

Political commitment and investment to control or eliminate malaria have contributed to an estimated 18% decrease in malaria incidence and a 25% decrease in malaria mortality between 2010 and 2016 [1]. Despite being largely preventable with affordable and easy-to-use treatment, malaria remains a major contributor to the global burden of disease among young children, representing the sixth leading cause of death among children aged <5 years globally [2–5]. In much of West Africa where malaria is holoendemic, the malaria burden increases seasonally during the annual rainy period [6]. This peak malaria season often coincides with the “hunger season,” the period before the annual harvest when household food stocks are limited and the risk of acute malnutrition in young children increases substantially [5, 7]. Many children in the region therefore may simultaneously suffer from both malaria and acute malnutrition.

While the common seasonality of malaria and malnutrition has been previously documented [8], the presence of a biological or immunological interaction between malaria and malnutrition that may encourage a high burden of both diseases remains unclear [9, 10]. It has been suggested that malnutrition may predispose children to infection or make it more difficult to recover from infection [8, 11–14]; however, the epidemiological evidence is mixed [15–25]. At the same time, malaria infection may predispose a child to acute weight loss or impair response to standard treatment for malnutrition [11]. Previous studies have shown malaria infection to be associated with poor nutritional status [26–30]. Heterogeneity in results may be due to differences in study populations, malaria transmission intensity, malaria diagnostic methods, growth metrics, indicators of growth and nutritional status, small sample sizes, or confounding by many interrelated factors.

To better understand the relationship between malaria and nutritional status, we used prospective data from a trial of children with severe acute malnutrition (SAM) [31]. We hypothesized that a bidirectional association exists, such that malnutrition increases the risk of malaria and malaria infection decreases the likelihood of nutritional recovery and response to nutritional treatment.

## METHODS

### Study Population

The present study is a secondary analysis of a randomized trial in Niger to assess the impact of routine antibiotic use in the outpatient treatment of uncomplicated SAM on nutritional recovery. The parent study was conducted at 4 rural health centers in the Madarounfa district of the Maradi region, Niger. Children who presented to 1 of the study centers for outpatient treatment for SAM were eligible for the parent study if they lived within 15 km of the center, were available to participate for the entire 12-week study period, had not been admitted to a nutritional program in the previous 3 months, and had not received an antibiotic within the previous 7 days. Eligible children further had no clinical complications that required antibiotic treatment and no congenital abnormalities. Outpatient treatment was provided for children aged 6–59 months with uncomplicated SAM, defined as weight-for-height Z-score (WHZ) less than −3 according to 2006 World Health Organization (WHO) growth standards and/or mid-upper arm circumference <115 mm, sufficient appetite by a test feeding of ready-to-use therapeutic food, and absence of clinical complications requiring inpatient treatment, including bipedal edema. There was no seasonal malaria chemoprophylaxis program in the study area during the year of follow-up.

Institutional review board approval for the parent study was obtained from the Comité Consultatif National d’Ethique, Niger, and the Comité de Protection des Personne, Île-de-France XI, Paris. Written informed consent was obtained from each child’s parent or legal guardian. An independent data and safety monitoring board reviewed study progress and safety events.

### Study Design and Procedures

Complete methods for the parent randomized, controlled trial have been previously reported [31]. In brief, children were randomized at admission for outpatient SAM treatment with a 1:1 allocation to receive amoxicillin or placebo for 7 days. Children were followed weekly until program discharge (minimum of 3 weeks) with additional scheduled study visits at weeks 4, 8, and 12. Children who were admitted to inpatient care were censored from follow-up but had vital status assessed 2 and 4 weeks after the date of transfer to inpatient care.

At admission, caregivers were asked about demographic and socioeconomic indicators, including the mother’s literacy, if the child had slept under a mosquito net the previous night, and number of children aged <5 years currently living in the household. Household resources were calculated via a principal components analysis that combined household resources, including number of televisions, radios, and livestock (eg, fowl, cows, and goats) belonging to the household. Caregivers reported on child illness in the last 24 hours (diarrhea, vomiting, and cough).

A rapid diagnostic histidine-rich protein 2 (HRP2)-based test for malaria (SD Bioline Malaria Antigen P.f, Standard Diagnostics Inc, Republic of Korea) was performed among all children at admission and among children with a temperature >38.5°C at any follow-up visit. Children with a positive rapid diagnostic test (RDT) received artemisinin-based combination therapy (ACT) (Coartem, Novartis) for the treatment of malaria. Hemoglobin concentration was measured on all children at admission (HemoCue Hb 301, HemoCue, Angelholm, Sweden). Weight, height, and mid-upper arm circumference were measured at all study visits, and anthropometric indices (eg, weight-for-height, height-for-age, and weight-for-age) were calculated according to 2006 WHO growth standards [32]. Indicators of dietary status at admission included household food security, dietary diversity, and current breastfeeding status (yes/no) and were assessed via caregiver report. Household food security was calculated as a sum of 13 questions related to household food resources in the previous 4 weeks, such as was there a time in the previous 4 weeks that the caregiver was concerned about not having enough food for the household, decreases in the number of meals in a day because of lack of resources, and going to bed hungry because there was not enough food to eat [33]. Dietary diversity was assessed by the number of 7 unique food groups consumed in the household in the last week, including [34] starch, vitamin A–rich foods, other fruits and vegetables, animal protein (eg, meat, eggs, poultry, fish), legumes, dairy, and fat (eg, oil, butter, other fat).

### Statistical Analyses

Baseline characteristics of the study population, overall and by malaria infection status at admission, were calculated with proportions for categorical variables and means and standard deviations (SDs) for continuous variables. The χ^2^ and *t* tests were used to compare differences in categorical and continuous variables, respectively, by malaria infection status at admission. To show temporal overlap in disease burden, malaria incidence and the total number of admissions to the nutritional program at the 4 health centers during the study period were calculated by month.

To assess the relationship between anthropometric indices and dietary status at admission with malaria incidence, we used Cox proportional hazards models. Anthropometric indices were time-updated, allowing for a more sensitive and proximal consideration of nutritional status. The outcome, that is, malaria incidence, was calculated among children who were malaria uninfected at admission, with person-time calculated from admission until the time of the first malaria infection or until administrative censoring at 12 weeks. Nonlinear associations with all anthropometric indices were assessed using splines and likelihood ratio tests.

Next, we assessed the relationship between malaria infection and the risk of nutritional recovery, time to recovery (days), and response to treatment, defined by weight gain (g/kg/day), height gain (mm/day), and change in anthropometric indices. Nutritional recovery at or before 8 weeks was defined as WHZ of −2 or greater on 2 consecutive visits and mid-upper arm circumference of 115 mm or greater. We used a Cox proportional hazards model to estimate the association of malaria infection at admission with the risk of nutrition recovery among all children, and we used linear regression for the association of malaria incidence with time to recovery (days) among children who recovered. The association of malaria infection at admission with indicators of response to treatment (eg, weight gain, height gain, and change in anthropometric indices) were modeled with linear mixed-effects models and adjusted for anthropometry at admission and time since admission. The potential dose–response relationship of multiple episodes of malaria during follow-up (eg, 0, 1, 2, 3 infections) on indicators of response to treatment was assessed using linear regression models. New infections were defined as presence of fever and an RDT positive at least 3 weeks after an earlier positive test, given the known time to negativity of rapid tests [35].

Final models were adjusted for potential confounders determined to be of a priori importance (age, sex, randomization arm in the parent trial [amoxicillin vs placebo], and site) and those identified with a univariate association of *P* < .30 [36]. All analyses were performed in Stata 14.1 (StataCorp, College Station, Texas).

## RESULTS

### Baseline Characteristics and Malaria Epidemiology

**Figure 1.**
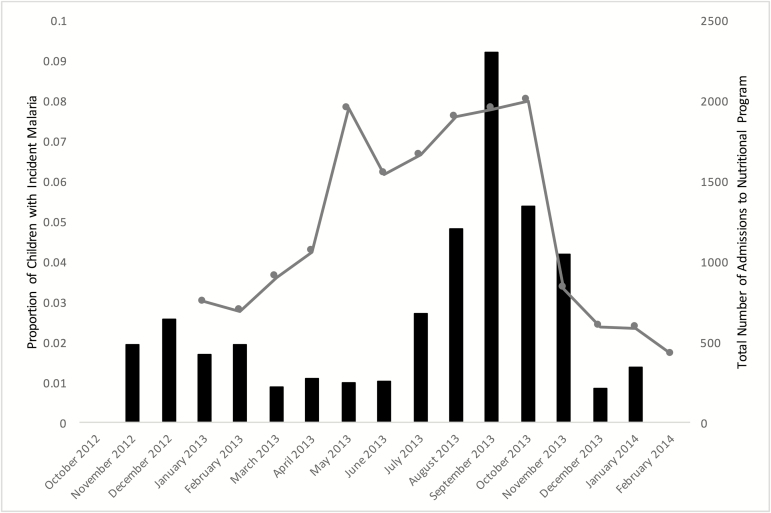
Histogram of malaria incidence and admissions to nutrition program. Black bars indicate the number of children during each month with incident malaria infection. The gray line indicates the number of admissions to the nutritional program per month.

Of 2399 children enrolled in the trial, 1327 (55.3%) were diagnosed with *Plasmodium falciparum* malaria at admission to SAM treatment. Table 1 displays descriptive characteristics at admission overall and among children with and without malaria infection at admission. Median age among those with malaria infection was 17.9 months (SD, 8.9 months) compared to 15.2 months among those without malaria (SD, 7.9 months; *P* < .001). Among children who did not have malaria infection at admission, incidence of first new malaria case over the follow-up period was 12.1 cases/100 person-months (95% confidence interval [CI], 11.1 to 13.1 cases/100 person-months). Cumulative incidence of at least 1 malaria infection was 30.1%. Higher malaria incidence temporally overlapped with the admissions to the nutritional program over the follow-up period (Figure 1).

**Table 1.** Baseline Descriptive Statistics of the Study Population

Characteristic	Overall Study Sample (N = 2399)	Malaria Uninfected at Admission (N = 1072)	Malaria Infected at Admission (N = 1327)	*P* Value^a^
Sociodemographic				
Age, m (mean, SD)	16.7 (8.6)	15.2 (7.9)	17.9 (8.9)	<.001
Female sex	1196 (49.9%)	508 (47.4%)	688 (51.9%)	.03
Mother is literate	474 (19.8%)	246 (23.0%)	228 (17.2%)	<.001
Household bednet use	2005 (83.6%)	852 (79.5%)	1153 (86.9%)	<.001
Number of children aged <5 years in household (mean, SD)	1.9 (1.2)	1.9 (1.2)	2.0 (1.3)	.06
Household assets (mean, SD)	0.001 (1.4)	−0.01 (1.3)	0.01 (1.5)	.71
Household food insecurity index (mean, SD)	8.2 (8.1)	8.3 (8.1)	8.2 (8.2)	.87
Dietary diversity score (mean, SD)	4.7 (1.5)	4.5 (1.6)	4.8 (1.5)	<.001
Currently breastfeeding aged <2 y	1496 (78.0%)	726 (78.3%)	770 (77.7%)	.11
Clinical and anthropometric				
Hemoglobin, g/dL (mean, SD)	9.6 (2.1)	10.6 (1.8)	8.8 (2.1)	<.001
Coughing	387 (16.1%)	157 (14.7%)	230 (17.3%)	.08
Vomiting, last 24 h	138 (5.8%)	58 (5.4%)	80 (6.0%)	.52
Diarrhea, last 24 h	759 (31.6%)	367 (34.2%)	392 (29.5%)	.01
Weight-for-height Z-score				
≥ −2	163 (6.8%)	82 (7.7%)	81 (6.1%)	…
< −2 and ≥ −3	904 (37.7%)	374 (34.9%)	530 (39.9%)	.22
< −3	1332 (55.5%)	616 (57.5%)	716 (54.0%)	…
Mean (SD)	–3.1 (0.64)	–3.1 (0.69)	–3.1 (0.60)	…
Height-for-age Z-score				
≥ –2	502 (20.9%)	247 (23.0%)	255 (19.2%)	…
< –2 and ≥ –3	734 (30.6%)	331 (30.9%)	403 (30.4%)	.07
< –3	1163 (48.5%)	494 (46.1%)	669 (50.4%)	…
Mean (SD)	–3.0 (1.2)	–2.9 (1.3)	–3.0 (1.2)	…
Weight-for-age Z-score				.98
≥ –2	9 (0.4%)	4 (0.4%)	5 (0.4%)	…
< –2 and ≥ –3	311 (13.0%)	149 (13.9%)	162 (12.2%)	…
< –3	2079 (86.7%)	909 (85.7%)	1160 (87.4%)	…
Mean (SD)	–3.8 (0.70)	–3.8 (0.72)	–3.8 (0.69)	…
Mid-upper arm circumference, cm				
<11.5	1869 (77.9%)	829 (77.3%)	1040 (78.4%)	…
11.5–12.4	528 (22.0%)	243 (22.7%)	285 (21.5%)	.10
12.5+	2 (0.08%)	0 (0%)	2 (0.2%)	…
Mean (SD)	11.2 (4.5)	112 (4.8)	112 (4.3)	…

Abbreviation: SD, standard deviation.

^a^χ^2^ test for categorical variables or t-test for continuous variables.

### Anthropometric Status and Dietary Indicators as Predictors of Malaria Incidence

No anthropometric index was associated with incident malaria infection over the follow-up period; however, children who were currently breastfeeding had 33% reduced hazards of malaria infection (Table 2 and Supplementary Appendix). Sensitivity analyses treating anthropometric indices with restricted cubic splines provided no evidence of a nonlinear association and did not change results. Results were qualitatively unchanged when stratified by age or restricted to children admitted to the nutritional program during the malaria season (Supplementary Table 1).

**Table 2.** Association Between Time-Updated Anthropometric Indices and Baseline Dietary Indicators With the Risk of First Malaria Infection During Severe Acute Malnutrition Treatment

Predictor	Unadjusted		Adjusted^a^	
	Hazards Ratio (95% CI)	*P* Trend	Adjusted Hazards Ratio (95% CI)	*P* Trend
Weight-for-height Z-score				
≥ –2	1.00	.51	1.00	.50
< –2 and ≥ –3	1.21 (0.95 to 1.55)	…	1.24 (0.96 to 1.59)	…
< –3	0.98 (0.66 to 1.44)	…	0.98 (0.66 to 1.45)	…
Height-for-age Z-score				
≥ –2	1.00	.13	1.00	.30
< –2 and ≥ –3	1.11 (0.80 to 1.53)	…	1.19 (0.85 to 1.65)	…
< –3	0.85 (0.63 to 1.15)	…	0.91 (0.67 to 1.25)	…
Weight-for-age Z-score				
≥ –2	1.00	.60	1.00	.90
< –2 and ≥ –3	0.96 (0.66 to 1.41)	…	0.96 (0.65 to 1.41)	…
< –3	0.92 (0.64 to 1.33)	…	1.00 (0.68 to 1.46)	…
Mid-upper arm circumference <11.5 cm	0.97 (0.75 to 1.27)	.85	0.99 (0.75 to 1.31)	.97
Household food insecurity index	1.00 (0.99 to 1.01)	.92	1.00 (0.98 to 1.01)	.66
Dietary diversity score	0.98 (0.91 to 1.04)	.47	0.95 (0.88 to 1.02)	.18
Current breastfeeding	0.83 (0.66 to 1.04)	.10	0.67 (0.47 to 0.96)	.03
Hemoglobin, g/dL	1.05 (0.99 to 1.11)	.09	1.01 (0.95 to 1.07)	.86

Abbreviations: CI, confidence interval.

^a^Adjusted for including child’s age and sex, mother’s literacy, number of children in the household aged <5 years, household bednet use, breastfeeding status, study site, calendar month, and cough, vomiting, diarrhea at admission.

### Malaria Infection as a Predictor of Nutritional Recovery and Treatment Response

Children diagnosed with malaria infection at admission and subsequently treated with ACT had increased risk of nutritional recovery compared to those who did not have malaria (adjusted hazards ratio, 1.16; 95% CI, 1.03 to 1.30; *P* = .01; Table 3 and Supplementary Appendix). Malaria infection at baseline was associated with all measures of increased weight and decreased height (Table 3 and Supplementary Appendix). Effects were stronger among children who were admitted during the malaria season compared to those admitted during the non-malaria season (Supplementary Table 2). There was no effect of multiple malaria episodes on nutritional recovery or growth outcomes (Supplementary Table 3).

**Table 3.** Association Between Malaria Infection at Admission With Nutritional Recovery (n = 2399) and Response to Treatment Among Children Who Recovered From Severe Acute Malnutrition (n = 1542)

Outcome	Univariate		Multivariable	
	Hazards Ratio	*P* Value	Adjusted Hazards Ratio	*P* Value
Nutritional recovery^a^	1.30 (1.18 to 1.44)	<.001	1.16 (1.03 to 1.30)	.01
	Mean (95% CI)		Adjusted mean (95% CI)	
Time until recovery (days)^a^	–1.21 (–2.25 to –0.18)	.02	–0.91 (–2.07 to 0.26)	.13
Mean weight change (kg)^b^	0.02 (–0.01 to 0.06)	.16	0.04 (0.004 to 0.08)	.03
Weight gain (g/kg/day)^b^	–0.02 (–0.28 to 0.25)	.89	0.38 (0.07 to 0.69)	.02
Mean height change (cm)^b^	–0.04 (–0.08 to –0.009)	.02	–0.07 (–0.10 to –0.03)	.001
Height change (mm/day)^b^	–0.001 (–0.003 to 0.0003)	.12	–0.002 (–0.004 to –0.0008)	.004
Weight-for-height Z-score ^b^	0.08 (0.04 to 0.13)	<.001	0.07 (0.02 to 0.12)	.005
Height-for-age Z-score^b^	0.005 (–0.01 to 0.02)	.55	–0.02 (–0.04 to –0.008)	.002
Weight-for-age Z-score^b^	0.05 (0.02 to 0.09)	.006	0.05 (0.009 to 0.09)	.02
Mid-upper arm circumference^b^	0.05 (0.01 to 0.09)	.008	0.03 (–0.01 to 0.07)	.20

Abbreviations: CI, confidence interval.

^a^Multivariable model adjusted for age at admission, sex, amoxicillin treatment arm, breastfeeding status, dietary diversity, mother’s literacy, mother’s age, site, and calendar month.

^b^Multivariable model adjusted for baseline, time since admission, age at admission, sex, amoxicillin treatment arm, breastfeeding status, dietary diversity, mother’s literacy, mother’s age, site, and calendar month.

## DISCUSSION

Consistent with reports of the epidemiology of malaria and malnutrition in Niger [5], we observed a strong seasonal pattern of both malaria incidence and admission to the therapeutic nutritional program, where half of children at admission presenting with a positive HRP2 RDT and the number of monthly incident malaria cases temporally overlapped with monthly admissions to the nutritional program. Contrary to our original hypotheses, we found that nutritional status was not associated with malaria incidence. However, treated malaria infection at baseline resulted in increased weight gain and decreased linear growth.

Cross-sectional studies have demonstrated an association between poor nutritional indicators and increased risk of malaria, suggesting that poor nutritional status might predispose children to malaria infection due to an impaired immune response associated with malnutrition [12, 13, 37–44]. However, results of prospective studies are mixed for the relationship between nutritional status and malaria incidence [15, 17–20, 25, 29, 45, 46]. Stunting, an indicator of chronic malnutrition that may be associated with an underlying impairment of the immune response [47], has been associated with both decreased [23, 48] and increased risks of malaria [15, 41]. The source of conflicting results for studies of malnourishment and malaria is unclear. There may be differences due to differences in populations under study, immunity, and/or differences in metrics used in studies that contribute to heterogeneity in results. In the present cohort, a lack of association between anthropometric indices and malaria incidence may have been attributable to the fact that all children in the study had SAM, and as such, there was less variability at admission.

Malaria infection in those treated with ACT was associated with increased weight gain and decreased height gain over 12 weeks compared to those without malaria infection. Weight gain effects during the malaria season were greater than in the non-malaria season. In the malaria season, children may be exposed to infected mosquito bites and may have subclinical infection that is not diagnosed by standard malaria RDT due to the low concentration of HRP2 in low parasitemia infections [49]. Such children (eg, those without RDT-positive malaria infection in the malaria season) would not benefit from effective treatment and fare relatively worse in terms of response to nutritional therapy. In addition, combination therapy in which partner drugs have long half-lives may provide a prophylactic effect and extended protection to support improved weight gain among treated children with malaria infection. All RDT-positive infections were treated in the present study, and it was not possible to disentangle the observed benefit of treatment on weight gain due to clearance of infection or prophylaxis against future infection.

We found a statistically significant effect of malaria infection on reduced height gain. The size of the effect was small, potentially due to the limited 12-week follow-up period, though height gain is also known to be generally limited in the context of nutritional rehabilitation, as linear growth in children may be curtailed until weight has stabilized [50]. In *P. falciparum* areas, previous studies have shown an association between baseline malaria infection and stunting [26–28], whereas others have shown no effect [29, 30]. A Mendelian randomization study that used the sickle cell trait as an instrumental variable for malaria infection status demonstrated a significant effect of repeated malaria infection on stunting [27]. By using a gene (sickle cell trait) as an instrument for exposure (malaria), this study removes some sources of confounding that are present in traditional observational studies.

Most dietary indicators at admission, including household food security and dietary diversity, were not associated with malaria incidence, but breastfeeding status was found to have a protective association. The mechanism for the protective effect of breastfeeding is unclear, but some evidence has shown antimalarial properties in breastmilk, including antibodies for *P. falciparum* and other breastmilk components that have antimalarial properties, such as lactoferrins and lipoproteins [51–53]. Nonbiological explanations, such as a breastfeeding child’s close contact with their mother providing protection from bites relative to children who are not breastfeeding, are also plausible and have been suggested elsewhere [54].

There are several strengths and limitations of this analysis. Strengths include the prospective nature of the data collection, with repeated measures. Data were collected as part of a randomized, controlled trial, and thus all outcomes were carefully standardized. Limitations of this analysis include that the study was restricted to children with SAM. As a result, there was a limited range of nutritional status at admission, which could limit the potential to explore a broader dose–response relationship. Comparison of children with SAM to those with mild or no malnutrition could provide additional important insights into the relationship between malaria and nutritional status. However, children with SAM represent a particularly vulnerable group with an increased risk of infectious illness and death, and these results provide important evidence for understanding the relationship between infection and nutritional status among these high-risk children. Second, assessment of malaria infection relied on HRP2 RDT, which has imperfect sensitivity for detecting malaria and may stay positive for several weeks following infection [35, 55, 56]. Although we used a 3-week period to define a new episode of malaria, it is possible that RDT positivity persisted longer than 3 weeks. In addition, RDTs during follow-up were only performed on children with fever, and as a result, some children with malaria but without fever may have been missed. Blood slides were not available in this study to confirm diagnosis. Future studies to assess the relationship between malaria and malnutrition could consider using microscopy to improve identification of malaria infection. Finally, follow-up was limited to 12 weeks, and the clinical significance of differences in linear growth during this shorter period of follow-up is not clear. Longer-term studies could determine whether this association persists and if the association is stronger over a longer period of time.

The results of this study demonstrate a potentially complex relationship between malnutrition and malaria. Nutritional status was not associated with incident malaria in children with uncomplicated SAM. Malaria infection treated with ACT resulted in increased weight and decreased height. Proper medical and nutritional management of malnourished children should be ensured to prevent adverse effects of malaria infection, including reduced height gain and linear growth faltering. Further study with longer follow-up may help elucidate the clinical significance of any effect of malaria infection and treatment on nutritional status.

## Supplementary Data

Supplementary materials are available at *Clinical Infectious Diseases* online. Consisting of data provided by the authors to benefit the reader, the posted materials are not copyedited and are the sole responsibility of the authors, so questions or comments should be addressed to the corresponding author.

Supplementary Appendix

Supplementary Tables

## Notes

***Financial Support.*** This study was supported by Médecins sans Frontières Operational Center Paris.

***Potential conflicts of interest.*** All authors: No reported conflicts of interest. All authors have submitted the ICMJE Form for Disclosure of Potential Conflicts of Interest. Conflicts that the editors consider relevant to the content of the manuscript have been disclosed.
