# Supplementary material for: Malaria and Nutritional Status Among Children With Severe Acute Malnutrition in Niger: A Prospective Cohort Study
Source: Clin Infect Dis. 2018 Mar 7;67(7):1027–34. doi: 10.1093/cid/ciy207 (PMC6137121; doi:10.1093/cid/ciy207)
Supplement: Supplementary Appendix [file ciy207_suppl_supplemental_appendix.docx]

**SUPPLEMENTAL APPENDIX**

This supplemental appendix provides details of unadjusted and adjusted models Table 2 and 3 in the main text.

**Table of Contents**

[Supplemental Appendix 1: Models for nutritional status predicting incident malaria 2](#_Toc507067492)

[**Supplemental Table 1.1.** Model for WHZ predicting incident malaria 2](#_Toc507067493)

[**Supplemental Table 1.2.** Model for HAZ predicting incident malaria 3](#_Toc507067494)

[**Supplemental Table 1.3.** Model for WAZ predicting incident malaria 4](#_Toc507067495)

[**Supplemental Table 1.4.** Model for MUAC predicting incident malaria 5](#_Toc507067496)

[**Supplemental Table 1.5.** Model for household food insecurity predicting incident malaria 6](#_Toc507067497)

[**Supplemental Table 1.6.** Model for dietary diversity predicting incident malaria 7](#_Toc507067498)

[**Supplemental Table 1.7.** Model for current breastfeeding predicting incident malaria 8](#_Toc507067499)

[**Supplemental Table 1.8.** Model for hemoglobin predicting incident malaria 9](#_Toc507067500)

[Supplemental Appendix 2: Models for baseline malaria infection predicting nutritional status 10](#_Toc507067501)

[**Supplemental Table 2.1.** Model for malaria predicting nutritional recovery 10](#_Toc507067502)

[**Supplemental Table 2.2.** Model for malaria predicting time until recovery 11](#_Toc507067503)

[**Supplemental Table 2.3.** Model for malaria predicting weight change 12](#_Toc507067504)

[**Supplemental Table 2.4.** Model for malaria predicting weight gain (g/kg/day) 13](#_Toc507067505)

[**Supplemental Table 2.5.** Model for malaria predicting height change 14](#_Toc507067506)

[**Supplemental Table 2.6.** Model for malaria predicting height change (mm/day) 15](#_Toc507067507)

[**Supplemental Table 2.7.** Model for malaria predicting WHZ 16](#_Toc507067508)

[**Supplemental Table 2.8.** Model for malaria predicting HAZ 17](#_Toc507067509)

[**Supplemental Table 2.9.** Model for malaria predicting WAZ 18](#_Toc507067510)

[**Supplemental Table 2.10.** Model for malaria predicting MUAC 19](#_Toc507067511)

# **Supplemental Appendix 1:** Models for nutritional status predicting incident malaria

This supplement includes tables including full details for each nutritional predictor of incident malaria, including both unadjusted and adjusted results for each covariate. All models are restricted to children without malaria at baseline (N=1,072).

## **Supplemental Table 1.1.** Model for WHZ predicting incident malaria

|  | **Unadjusted** | | **Adjusted^1^** | |
| --- | --- | --- | --- | --- |
|  | *HR (95% CI)* | *P-trend* | *aHR (95% CI)^1^* | *P-trend* |
| WHZ  ≥-2  <-2 and ≥-3  < -3 | 1.00  1.21 (0.95 to 1.55)  0.98 (0.66 to 1.44) | 0.51 | 1.00  1.24 (0.96 to 1.59)  0.98 (0.66 to 1.45) | 0.50 |
| Breastfeeding | 0.83 (0.66 to 1.04) | 0.10 | 0.65 (0.45 to 0.94) | 0.02 |
| Bednet use | 0.50 (0.37 to 0.67) | <0.001 | 0.78 (0.58 to 1.06) | 0.12 |
| Mother literate | 1.39 (1.08 to 1.80) | 0.01 | 1.02 (0.78 to 1.34) | 0.88 |
| Child’s age | 1.00 (0.99 to 1.01) | 0.92 | 0.98 (0.95 to 1.00) | 0.05 |
| Child’s sex | 0.87 (0.70 to 1.09) | 0.22 | 0.9 2(0.74 to 1.16) | 0.49 |
| Number of children in household <5 | 1.16 (1.07 to 1.25) | <0.001 | 1.08 (0.98 to 1.18) | 0.11 |
| Coughing | 1.26 (0.85 to 1.86) | 0.24 | 0.89 (0.58 to 1.36) | 0.59 |
| Vomiting | 1.58 (0.98 to 2.54) | 0.06 | 1.29 (0.79 to 2.10) | 0.31 |
| Diarrhea | 1.17 (0.94 to 1.46) | 0.17 | 1.11 (0.87 to 1.41) | 0.41 |
| Study site  1  2  3  4 | Ref  0.61 (0.44 to 0.84)  0.84 (0.59 to 1.19)  0.43 (0.33 to 0.56) | 0.003  0.33  <0.001 | Ref  0.77 (0.55 to 1.08)  0.84 (0.57 to 1.23)  0.54 (0.41 to 0.72) | 0.13  0.36  <0.001 |
| Admission month | 0.28 (0.22 to 0.36) | <0.001 | 0.56 (0.42 to 0.75) | <0.001 |

^1^Adjusted for including child’s age and sex, mother’s literacy, number of children in the household under the age of 5, household bednet use, breastfeeding status, study site, calendar month, and cough, vomiting, diarrhea at admission.

## **Supplemental Table 1.2.** Model for HAZ predicting incident malaria

|  | **Unadjusted** | | **Adjusted^1^** | |
| --- | --- | --- | --- | --- |
|  | *HR (95% CI)* | *P-trend* | *aHR (95% CI)^1^* | *P-trend* |
| HAZ  ≥-2  <-2 and ≥-3  < -3 | 1.00  1.11 (0.80 to 1.53)  0.85 (0.63 to 1.15) | 0.13 | 1.00  1.19 (0.85 to 1.65)  0.91 (0.67 to 1.25) | 0.30 |
| Breastfeeding | 0.83 (0.66 to 1.04) | 0.10 | 0.66 (0.65 to 0.95) | 0.03 |
| Bednet use | 0.50 (0.37 to 0.67) | <0.001 | 0.79 (0.58 to 1.08) | 0.14 |
| Mother literate | 1.39 (1.08 to 1.80) | 0.01 | 1.01 (0.77 to 1.33) | 0.92 |
| Child’s age | 1.00 (0.99 to 1.01) | 0.92 | 0.98 (0.96 to 1.00) | 0.10 |
| Child’s sex | 0.87 (0.70 to 1.09) | 0.22 | 0.90 (0.72 to 1.13) | 0.37 |
| Number of children in household <5 | 1.16 (1.07 to 1.25) | <0.001 | 1.09 (0.99 to 1.19) | 0.07 |
| Coughing | 1.26 (0.85 to 1.86) | 0.24 | 0.89 (0.58 to 1.36) | 0.59 |
| Vomiting | 1.58 (0.98 to 2.54) | 0.06 | 1.30 (0.80 to 2.13) | 0.29 |
| Diarrhea | 1.17 (0.94 to 1.46) | 0.17 | 1.08 (0.85 to 1.37) | 0.54 |
| Study site  1  2  3  4 | Ref  0.61 (0.44 to 0.84)  0.84 (0.59 to 1.19)  0.43 (0.33 to 0.56) | 0.003  0.33  <0.001 | Ref  0.76 (0.54 to 1.05)  0.82 (0.56 to 1.20)  0.56 (0.42 to 0.75) | 0.10  0.31  <0.001 |
| Admission month | 0.28 (0.22 to 0.36) | <0.001 | 0.55 (0.41 to 0.74) | <0.001 |

^1^Adjusted for including child’s age and sex, mother’s literacy, number of children in the household under the age of 5, household bednet use, breastfeeding status, study site, calendar month, and cough, vomiting, diarrhea at admission.

## **Supplemental Table 1.3.** Model for WAZ predicting incident malaria

|  | **Unadjusted** | | **Adjusted^1^** | |
| --- | --- | --- | --- | --- |
|  | *HR (95% CI)* | *P-trend* | *aHR (95% CI)^1^* | *P-trend* |
| WAZ  ≥-2  <-2 and ≥-3  < -3 | 1.00  0.96 (0.66 to 1.41)  0.92 (0.64 to 1.33) | 0.60 | 1.00  0.96 (0.65 to 1.41)  1.00 (0.68 to 1.46) | 0.90 |
| Breastfeeding | 0.83 (0.66 to 1.04) | 0.10 | 0.65 (0.45 to 0.95) | 0.03 |
| Bednet use | 0.50 (0.37 to 0.67) | <0.001 | 0.78 (0.58 to 1.07) | 0.12 |
| Mother literate | 1.39 (1.08 to 1.80) | 0.01 | 1.02 (0.78 to 1.33) | 0.90 |
| Child’s age | 1.00 (0.99 to 1.01) | 0.92 | 0.98 (0.95 to 1.00) | 0.05 |
| Child’s sex | 0.87 (0.70 to 1.09) | 0.22 | 0.92 (0.73 to 1.15) | 0.46 |
| Number of children in household <5 | 1.16 (1.07 to 1.25) | <0.001 | 1.08 (0.99 to 1.18) | 0.10 |
| Coughing | 1.26 (0.85 to 1.86) | 0.24 | 0.88 (0.58 to 1.35) | 0.57 |
| Vomiting | 1.58 (0.98 to 2.54) | 0.06 | 1.26 (0.77 to 2.05) | 0.37 |
| Diarrhea | 1.17 (0.94 to 1.46) | 0.17 | 1.10 (0.86 to 1.40) | 0.45 |
| Study site  1  2  3  4 | Ref  0.61 (0.44 to 0.84)  0.84 (0.59 to 1.19)  0.43 (0.33 to 0.56) | 0.003  0.33  <0.001 | Ref  0.76 (0.54 to 1.06)  0.81 (0.55 to 1.18)  0.55 (0.41 to 0.73) | 0.11  0.28  <0.001 |
| Admission month | 0.28 (0.22 to 0.36) | <0.001 | 0.55 (0.41 to 0.74) | <0.001 |

^1^Adjusted for including child’s age and sex, mother’s literacy, number of children in the household under the age of 5, household bednet use, breastfeeding status, study site, calendar month, and cough, vomiting, diarrhea at admission.

## **Supplemental Table 1.4.** Model for MUAC predicting incident malaria

|  | **Unadjusted** | | **Adjusted^1^** | |
| --- | --- | --- | --- | --- |
|  | *HR (95% CI)* | *P* | *aHR (95% CI)^1^* | *P* |
| MUAC <115 | 0.97 (0.75 to 1.27) | 0.85 | 0.99 (0.75 to 1.31) | 0.97 |
| Breastfeeding | 0.83 (0.66 to 1.04) | 0.10 | 0.66 (0.46 to 0.95) | 0.03 |
| Bednet use | 0.50 (0.37 to 0.67) | <0.001 | 0.79 (0.58 to 1.07) | 0.13 |
| Mother literate | 1.39 (1.08 to 1.80) | 0.01 | 1.02 (0.78 to 1.34) | 0.90 |
| Child’s age | 1.00 (0.99 to 1.01) | 0.92 | 0.98 (0.95 to 1.00) | 0.05 |
| Child’s sex | 0.87 (0.70 to 1.09) | 0.22 | 0.92 (0.73 to 1.15) | 0.45 |
| Number of children in household <5 | 1.16 (1.07 to 1.25) | <0.001 | 1.08 (0.99 to 1.18) | 0.10 |
| Coughing | 1.26 (0.85 to 1.86) | 0.24 | 0.88 (0.58 to 1.35) | 0.56 |
| Vomiting | 1.58 (0.98 to 2.54) | 0.06 | 1.26 (0.78 to 2.05) | 0.35 |
| Diarrhea | 1.17 (0.94 to 1.46) | 0.17 | 1.09 (0.86 to 1.39) | 0.46 |
| Study site  1  2  3  4 | Ref  0.61 (0.44 to 0.84)  0.84 (0.59 to 1.19)  0.43 (0.33 to 0.56) | 0.003  0.33  <0.001 | Ref  0.75 (0.54 to 1.06)  0.81 (0.55 to 1.19)  0.55 (0.41 to 0.74) | 0.11  0.28  <0.001 |
| Admission month | 0.28 (0.22 to 0.36) | <0.001 | 0.55 (0.41 to 0.74) | <0.001 |

^1^Adjusted for including child’s age and sex, mother’s literacy, number of children in the household under the age of 5, household bednet use, breastfeeding status, study site, calendar month, and cough, vomiting, diarrhea at admission.

## **Supplemental Table 1.5.** Model for household food insecurity predicting incident malaria

|  | **Unadjusted** | | **Adjusted^1^** | |
| --- | --- | --- | --- | --- |
|  | *HR (95% CI)* | *P* | *aHR (95% CI)^1^* | *P* |
| Household food insecurity | 1.00 (0.99 to 1.01) | 0.92 | 1.00 (0.98 to 1.01) | 0.66 |
| Breastfeeding | 0.83 (0.66 to 1.04) | 0.10 | 0.67 (0.47 to 0.97) | 0.03 |
| Bednet use | 0.50 (0.37 to 0.67) | <0.001 | 0.69 (0.51 to 0.94) | 0.02 |
| Mother literate | 1.39 (1.08 to 1.80) | 0.01 | 1.04 (0.80 to 1.37) | 0.75 |
| Child’s age | 1.00 (0.99 to 1.01) | 0.92 | 0.98 (0.96 to 1.01) | 0.14 |
| Child’s sex | 0.87 (0.70 to 1.09) | 0.22 | 0.90 (0.72 to 1.13) | 0.37 |
| Number of children in household <5 | 1.16 (1.07 to 1.25) | <0.001 | 1.11 (1.02 to 1.21) | 0.02 |
| Coughing | 1.26 (0.85 to 1.86) | 0.24 | 0.96 (0.64 to 1.44) | 0.84 |
| Vomiting | 1.58 (0.98 to 2.54) | 0.06 | 1.41 (0.87 to 2.29) | 0.17 |
| Diarrhea | 1.17 (0.94 to 1.46) | 0.17 | 1.16 (0.91 to 1.47) | 0.24 |
| Study site  1  2  3  4 | Ref  0.61 (0.44 to 0.84)  0.84 (0.59 to 1.19)  0.43 (0.33 to 0.56) | 0.003  0.33  <0.001 | Ref  0.71 (0.51 to 0.99)  0.82 (0.56 to 1.21)  0.51 (0.38 to 0.68) | 0.04  0.33  <0.001 |
| Admission month | 0.28 (0.22 to 0.36) | <0.001 | 0.29 (0.22 to 0.38) | <0.001 |

^1^Adjusted for including child’s age and sex, mother’s literacy, number of children in the household under the age of 5, household bednet use, breastfeeding status, study site, calendar month, and cough, vomiting, diarrhea at admission.

## **Supplemental Table 1.6.** Model for dietary diversity predicting incident malaria

|  | **Unadjusted** | | **Adjusted^1^** | |
| --- | --- | --- | --- | --- |
|  | *HR (95% CI)* | *P* | *aHR (95% CI)^1^* | *P* |
| Dietary diversity | 0.98 (0.91 to 1.04) | 0.47 | 0.95 (0.88 to 1.02) | 0.18 |
| Breastfeeding | 0.83 (0.66 to 1.04) | 0.10 | 0.65 (0.45 to 0.94) | 0.02 |
| Bednet use | 0.50 (0.37 to 0.67) | <0.001 | 0.70 (0.52 to 0.95) | 0.02 |
| Mother literate | 1.39 (1.08 to 1.80) | 0.01 | 1.07 (0.82 to 1.40) | 0.62 |
| Child’s age | 1.00 (0.99 to 1.01) | 0.92 | 0.98 (0.96 to 1.01) | 0.15 |
| Child’s sex | 0.87 (0.70 to 1.09) | 0.22 | 0.91 (0.73 to 1.14) | 0.41 |
| Number of children in household <5 | 1.16 (1.07 to 1.25) | <0.001 | 1.11 (1.01 to 1.21) | 0.02 |
| Coughing | 1.26 (0.85 to 1.86) | 0.24 | 0.95 (0.63 to 1.43) | 0.81 |
| Vomiting | 1.58 (0.98 to 2.54) | 0.06 | 1.41 (0.87 to 2.30) | 0.17 |
| Diarrhea | 1.17 (0.94 to 1.46) | 0.17 | 1.16 (0.91 to 1.47) | 0.23 |
| Study site  1  2  3  4 | Ref  0.61 (0.44 to 0.84)  0.84 (0.59 to 1.19)  0.43 (0.33 to 0.56) | 0.003  0.33  <0.001 | Ref  0.72 (0.52 to 1.01)  0.81 (0.56 to 1.19)  0.49 (0.36 to 0.65) | 0.06  0.29  <0.001 |
| Admission month | 0.28 (0.22 to 0.36) | <0.001 | 0.29 (0.23 to 0.38) | <0.001 |

^1^Adjusted for including child’s age and sex, mother’s literacy, number of children in the household under the age of 5, household bednet use, breastfeeding status, study site, calendar month, and cough, vomiting, diarrhea at admission.

## **Supplemental Table 1.7.** Model for current breastfeeding predicting incident malaria

|  | **Unadjusted** | | **Adjusted^1^** | |
| --- | --- | --- | --- | --- |
|  | *HR (95% CI)* | *P* | *aHR (95% CI)^1^* | *P* |
| Current breastfeeding | 0.83 (0.66 to 1.04) | 0.10 | 0.67 (0.47 to 0.96) | 0.03 |
| Bednet use | 0.50 (0.37 to 0.67) | <0.001 | 0.69 (0.51 to 0.94) | 0.02 |
| Mother literate | 1.39 (1.08 to 1.80) | 0.01 | 1.05 (0.80 to 1.37) | 0.73 |
| Child’s age | 1.00 (0.99 to 1.01) | 0.92 | 0.98 (0.96 to 1.01) | 0.13 |
| Child’s sex | 0.87 (0.70 to 1.09) | 0.22 | 0.90 (0.72 to 1.13) | 0.37 |
| Number of children in household <5 | 1.16 (1.07 to 1.25) | <0.001 | 1.11 (1.02 to 1.21) | 0.02 |
| Coughing | 1.26 (0.85 to 1.86) | 0.24 | 0.96 (0.64 to 1.45) | 0.85 |
| Vomiting | 1.58 (0.98 to 2.54) | 0.06 | 1.40 (0.86 to 2.29) | 0.17 |
| Diarrhea | 1.17 (0.94 to 1.46) | 0.17 | 1.16 (0.91 to 1.47) | 0.23 |
| Study site  1  2  3  4 | Ref  0.61 (0.44 to 0.84)  0.84 (0.59 to 1.19)  0.43 (0.33 to 0.56) | 0.003  0.33  <0.001 | Ref  0.71 (0.51 to 0.99)  0.81 (0.56 to 1.19)  0.51 (0.38 to 0.67) | 0.04  0.28  <0.001 |
| Admission month | 0.28 (0.22 to 0.36) | <0.001 | 0.29 (0.23 to 0.38) | <0.001 |

^1^Adjusted for including child’s age and sex, mother’s literacy, number of children in the household under the age of 5, household bednet use, breastfeeding status, study site, calendar month, and cough, vomiting, diarrhea at admission.

## **Supplemental Table 1.8.** Model for hemoglobin predicting incident malaria

|  | **Unadjusted** | | **Adjusted^1^** | |
| --- | --- | --- | --- | --- |
|  | *HR (95% CI)* | *P* | *aHR (95% CI)^1^* | *P* |
| Hemoglobin | 1.05 (0.99 to 1.11) | 0.09 | 1.01 (0.95 to 1.07) | 0.86 |
| Breastfeeding | 0.83 (0.66 to 1.04) | 0.10 | 0.67 (0.47 to 0.96) | 0.03 |
| Bednet use | 0.50 (0.37 to 0.67) | <0.001 | 0.69 (0.51 to 0.94) | 0.02 |
| Mother literate | 1.39 (1.08 to 1.80) | 0.01 | 1.05 (0.80 to 1.37) | 0.74 |
| Child’s age | 1.00 (0.99 to 1.01) | 0.92 | 0.98 (0.96 to 1.01) | 0.14 |
| Child’s sex | 0.87 (0.70 to 1.09) | 0.22 | 0.90 (0.72 to 1.13) | 0.38 |
| Number of children in household <5 | 1.16 (1.07 to 1.25) | <0.001 | 1.11 (1.02 to 1.21) | 0.02 |
| Coughing | 1.26 (0.85 to 1.86) | 0.24 | 0.96 (0.64 to 1.45) | 0.85 |
| Vomiting | 1.58 (0.98 to 2.54) | 0.06 | 1.40 (0.86 to 2.28) | 0.17 |
| Diarrhea | 1.17 (0.94 to 1.46) | 0.17 | 1.16 (0.91 to 1.47) | 0.24 |
| Study site  1  2  3  4 | Ref  0.61 (0.44 to 0.84)  0.84 (0.59 to 1.19)  0.43 (0.33 to 0.56) | 0.003  0.33  <0.001 | Ref  0.72 (0.51 to 1.01)  0.83 (0.54 to 1.26)  0.51 (0.38 to 0.68) | 0.06  0.38  <0.001 |
| Admission month | 0.28 (0.22 to 0.36) | <0.001 | 0.29 (0.23 to 0.38) | <0.001 |

^1^Adjusted for including child’s age and sex, mother’s literacy, number of children in the household under the age of 5, household bednet use, breastfeeding status, study site, calendar month, and cough, vomiting, diarrhea at admission.

# **Supplemental Appendix 2:** Models for baseline malaria infection predicting nutritional status

This supplement includes tables including full details for each model of baseline malaria predicting nutritional outcomes.

## **Supplemental Table 2.1.** Model for malaria predicting nutritional recovery

|  | **Univariate** | | **Multivariable^1^** | |
| --- | --- | --- | --- | --- |
|  | HR | *P* | aHR | *P* |
| Malaria | 1.30 (1.18 to 1.44) | <0.001 | 1.16 (1.03 to 1.30) | 0.01 |
| Child’s age | 1.02 (1.01 to 1.02) | <0.001 | 1.01 (1.00 to 1.02) | 0.16 |
| Child’s sex | 1.21 (1.09 to 1.33) | <0.001 | 1.23 (1.11 to 1.36) | <0.001 |
| Baseline hemoglobin | 0.96 (0.94 to 0.99) | 0.003 | 1.00 (0.97 to 1.03) | 0.90 |
| Amoxicillin treatment arm | 1.09 (0.98 to 1.20) | 0.11 | 1.10 (0.99 to 1.21) | 0.07 |
| Breastfeeding | 0.72 (0.65 to 0.80) | <0.001 | 0.79 (0.67 to 0.92) | 0.003 |
| Dietary diversity | 1.09 (1.05 to 1.13) | <0.001 | 1.05 (1.02 to 1.09) | 0.005 |
| Mother literate | 0.93 (0.82 to 1.06) | 0.27 | 0.95 (0.83 to 1.09) | 0.50 |
| Food security | 1.00 (0.99 to 1.01) | 0.90 | 1.00 (0.99 to 1.01) | 0.91 |
| Site  1  2  3  4 | Ref  1.07 (0.94 to 1.23)  1.27 (1.11 to 1.47)  0.84 (0.73 to 0.97) | 0.29  0.001  0.02 | Ref  1.02 (0.89 to 1.17)  1.30 (1.12 to 1.50)  0.80 (0.69 to 0.93) | 0.79  <0.001  0.003 |
| Admission month | 1.03 (1.01 to 1.05) | 0.001 | 1.03 (1.01 to 1.05) | <0.001 |

^1^Adjusted for admission weight and time since admission; ^2^Adjusted for admission weight, time since admission, age at admission, sex, amoxicillin treatment arm, breastfeeding status, dietary diversity, mother’s literacy, mother’s age, site, and calendar month

## **Supplemental Table 2.2.** Model for malaria predicting time until recovery

|  | **Univariate** | | **Multivariable^1^** | |
| --- | --- | --- | --- | --- |
|  | *Mean (95% CI)* | *P* | *Adjusted Mean*  *(95% CI)* | *P* |
| Malaria | -1.21 (-2.25 to -0.18) | 0.02 | -0.91 (-2.07 to 0.26) | 0.13 |
| Child’s age | -0.14 (-0.20 to -0.08) | <0.001 | -0.05 (-0.14 to 0.05) | 0.32 |
| Child’s sex | -1.68 (-2.69 to -0.66) | 0.001 | -1.97 (-2.97 to -0.96) | <0.001 |
| Baseline hemoglobin | 0.12 (-0.11 to 0.36) | 0.31 | -0.04 (-0.30 to 0.23) | 0.78 |
| Amoxicillin treatment arm | -1.88 (-2.90 to -0.86) | <0.001 | -1.89 (-2.90 to -0.89) | <0.001 |
| Breastfeeding | 2.75 (1.72 to 3.78) | <0.001 | 2.22 (0.58 to 3.86) | 0.008 |
| Dietary diversity | -0.37 (-0.72 to -0.02) | 0.04 | -0.15 (-0.52 to 0.22) | 0.43 |
| Mother literate | -0.44 (-1.76 to 0.88) | 0.51 | -0.46 (-1.79 to 0.88) | 0.50 |
| Food security | -0.0009 (-0.06 to 0.06) | 0.98 | 0.008 (-0.05 to 0.07) | 0.81 |
| Site  1  2  3  4 | Ref  -0.17 (-1.54 to 1.19)  -1.83 (-3.26 to -0.40)  1.49 (0.08 to 2.90) | 0.80  0.01  0.04 | Ref  -0.13 (-1.53 to 1.28)  -2.35 (-3.80 to -0.91) 1.32 (-0.14 to 2.79) | 0.86  0.001  0.08 |
| Admission month | 0.007 (-0.17 to 0.18) | 0.94 | -0.02 (-0.20 to 0.16) | 0.79 |

^1^Adjusted for admission weight and time since admission; ^2^Adjusted for admission weight, time since admission, age at admission, sex, amoxicillin treatment arm, breastfeeding status, dietary diversity, mother’s literacy, mother’s age, site, and calendar month.

## **Supplemental Table 2.3.** Model for malaria predicting weight change

|  | **Univariate** | | **Multivariable^1^** | |
| --- | --- | --- | --- | --- |
|  | *Mean (95% CI)* | *P* | *Adjusted Mean*  *(95% CI)* | *P* |
| Malaria | 0.02 (-0.01 to 0.06) | 0.16 | 0.04 (0.004 to 0.08) | 0.03 |
| Child’s age | 0.02 (0.01 to 0.02) | <0.001 | 0.02 (0.01 to 0.02) | <0.001 |
| Child’s sex | -0.07 (-0.10 to -0.04) | <0.001 | -0.12 (-0.15 to -0.09) | <0.001 |
| Baseline hemoglobin | 0.003 (-0.005 to 0.01) | 0.48 | 0.007 (-0.002 to 0.01) | 0.12 |
| Amoxicillin treatment arm | 0.10 (0.06 to 0.13) | <0.001 | 0.09 (0.06 to 0.13) | <0.001 |
| Breastfeeding | -0.20 (-0.24 to -0.15) | <0.001 | -0.10 (-0.16 to -0.05) | <0.001 |
| Dietary diversity | 0.01 (0.002 to 0.02) | 0.02 | 0.01 (0.002 to 0.02) | 0.02 |
| Mother literate | -0.007 (-0.05 to 0.04) | 0.76 | -0.001 (0.04 to 0.04) | 0.95 |
| Site  1  2  3  4 | Ref  0.08 (0.03 to 0.12)  0.02 (-0.02 to 0.07)  0.009 (-0.04 to 0.06) | 0.001  0.32  0.69 | Ref  0.06 (0.02 to 0.10)  0.02 (-0.02 to 0.07)  -0.02 (-0.07 to 0.02) | 0.007  0.35  0.32 |
| Admission month | 0.002 (-0.0007 to 0.006) |  | 0.003 (-0.0006 to 0.006) | 0.11 |

^1^Adjusted for admission weight and time since admission; ^2^Adjusted for admission weight, time since admission, age at admission, sex, amoxicillin treatment arm, breastfeeding status, dietary diversity, mother’s literacy, mother’s age, site, and calendar month.

## **Supplemental Table 2.4.** Model for malaria predicting weight gain (g/kg/day)

|  | **Univariate** | | **Multivariable^1^** | |
| --- | --- | --- | --- | --- |
|  | *Mean (95% CI)* | *P* | *Adjusted Mean*  *(95% CI)* | *P* |
| Malaria | -0.02 (-0.28 to 0.25) | 0.89 | 0.38 (0.07 to 0.69) | 0.02 |
| Child’s age | 0.12 (0.10 to 0.15) | <0.001 | 0.11 (0.08 to 0.14) | <0.001 |
| Child’s sex | -0.50 (-0.79 to -0.21) | <0.001 | -0.87 (-1.16 to -0.59) | <0.001 |
| Baseline hemoglobin | 0.07 (-0.001 to 0.13) | 0.06 | 0.09 (0.02 to 0.16) | 0.01 |
| Amoxicillin treatment arm | 1.10 (0.82 to 1.38) | <0.001 | 1.07 (0.81 to 1.34) | <0.001 |
| Breastfeeding | -1.72 (-2.11 to -1.33) | <0.001 | -1.07 (-1.51 to -0.63) | <0.001 |
| Dietary diversity | 0.10 (0.02 to 0.18) | 0.01 | 0.09 (0.02 to 0.17) | 0.01 |
| Mother literate | 0.10 (-0.27 to 0.47) | 0.61 | 0.12 (-0.24 to 0.47) | 0.52 |
| Site  1  2  3  4 | Ref  0.57 (0.19 to 0.96)  0.42 (0.02 to 0.82)  0.11 (-0.29 to 0.50) | 0.003  0.04  0.60 | Ref  0.40 (0.05 to 0.77)  0.42 (0.04 to 0.80)  -0.07 (-0.46 to 0.32) | 0.03  0.03  0.73 |
| Admission month | 0.002 (-0.03 to 0.04) | 0.89 | 0.004 (-0.03 to 0.04) | 0.80 |

^1^Adjusted for admission weight and time since admission; ^2^Adjusted for admission weight, time since admission, age at admission, sex, amoxicillin treatment arm, breastfeeding status, dietary diversity, mother’s literacy, mother’s age, site, and calendar month.

## **Supplemental Table 2.5.** Model for malaria predicting height change

|  | **Univariate** | | **Multivariable^1^** | |
| --- | --- | --- | --- | --- |
|  | *Mean (95% CI)* | *P* | *Adjusted Mean*  *(95% CI)* | *P* |
| Malaria | -0.04 (-0.08 to -0.009) | 0.02 | -0.07 (-0.10 to -0.03) | 0.001 |
| Child’s age | 0.009 (-0.003 to 0.005) | 0.61 | 0.003 (-0.001 to 0.007) | 0.19 |
| Child’s sex | -0.02 (-0.05 to 0.02) | 0.30 | -0.01 (-0.04 to 0.02) | 0.56 |
| Baseline hemoglobin | -0.0008 (-0.009 to 0.007) | 0.85 | -0.01 (-0.02 to -0.002) | 0.02 |
| Amoxicillin treatment arm | 0.005 (-0.03 to 0.04) | 0.78 | 0.006 (-0.03 to 0.04) | 0.72 |
| Breastfeeding | -0.008 (-0.06 to 0.04) | 0.76 | 002 (-0.04 to 0.07) | 0.60 |
| Dietary diversity | -0.002 (-0.01 to 0.008) | 0.75 | -0.008 (-0.02 to 0.001) | 0.09 |
| Mother literate | -0.04 (-0.08 to 0.009) | 0.012 | -0.03 (-0.07 to 0.01) | 0.18 |
| Site  1  2  3  4 | Ref  0.10 (0.06 to 0.15)  0.14 (0.09 to 0.19) -0.07 (-0.12 to -0.02) | <0.001  <0.001  0.003 | Ref  0.12 (0.07 to 0.16)  0.14 (0.09 to 0.19)  -0.06 (-0.11 to -0.01) | <0.001  <0.001  0.01 |
| Admission month | -0.01 (-0.02 to -0.009) | <0.001 | -0.01 (-0.02 to -0.006) | <0.001 |

^1^Adjusted for admission weight and time since admission; ^2^Adjusted for admission weight, time since admission, age at admission, sex, amoxicillin treatment arm, breastfeeding status, dietary diversity, mother’s literacy, mother’s age, site, and calendar month.

## **Supplemental Table 2.6.** Model for malaria predicting height change (mm/day)

|  | **Univariate** | | **Multivariable^1^** | |
| --- | --- | --- | --- | --- |
|  | *Mean (95% CI)* | *P* | *Adjusted Mean*  *(95% CI)* | *P* |
| Malaria | -0.001 (-0.003 to 0.0003) | 0.12 | -0.002 (-0.004 to -0.0008) | 0.004 |
| Child’s age | 0.00001 (-0.0001 to 0.0002) | 0.89 | 0.00006 (-0.0001 to 0.0002) | 0.55 |
| Child’s sex | -0.0008 (-0.002 to 0.0007) | 0.30 | -0.0006 (-0.002 to 0.001) | 0.55 |
| Baseline hemoglobin | -0.00004 (-0.0003 to 0.0003) | 0.82 | -0.0004 (-0.0008 to 0) | 0.05 |
| Amoxicillin treatment arm | -0.00009 (-0.002 to 0.001) | 0.90 | -0.00007 (-0.002 to 0.001) | 0.93 |
| Breastfeeding | -0.0002 (-0.002 to 0.002) | 0.87 | 0.0005 (-0.002 to 0.003) | 0.71 |
| Dietary diversity | 0.00002 (-0.0004 to 0.0004) | 0.92 | -0.0002 (-0.0006 to 0.0003) | 0.46 |
| Mother literate | -0.001 (-0.003 to 0.0005) | 0.16 | -0.001 (-0.003 to 0.0008) | 0.23 |
| Site  1  2  3  4 | Ref  0.004 (0.002 to 0.006)  0.003 (0.001 to 0.005)  -0.0009 (-0.003 to 0.001) | <0.001  0.002  0.38 | Ref  0.004 (0.002 to 0.006)  0.003 (0.001 to 0.005)  -0.0007 (-0.003 to 0.001) | <0.001  0.003  0.51 |
| Admission month | -0.0004 (-0.0006 to -0.0002) | <0.001 | -0.0003 (-0.0005 to -0.001) | 0.001 |

^1^Adjusted for admission weight and time since admission; ^2^Adjusted for admission weight, time since admission, age at admission, sex, amoxicillin treatment arm, breastfeeding status, dietary diversity, mother’s literacy, mother’s age, site, and calendar month.

## **Supplemental Table 2.7.** Model for malaria predicting WHZ

|  | **Univariate** | | **Multivariable^1^** | |
| --- | --- | --- | --- | --- |
|  | *Mean (95% CI)* | *P* | *Adjusted Mean*  *(95% CI)* | *P* |
| Malaria | 0.08 (0.04 to 0.13) | <0.001 | 0.07 (0.02 to 0.12) | 0.005 |
| Child’s age | 0.009 (0.007 to 0.01) | <0.001 | 0.005 (0.0005 to 0.009) | 0.03 |
| Child’s sex | -0.05 (-0.10 to -0.002) | 0.04 | -0.06 (-0.10 to 0.008) | 0.02 |
| Baseline hemoglobin | -0.01 (-0.02 to 0.001) | 0.08 | 0.003 (-0.008 to 0.02) | 0.58 |
| Amoxicillin treatment arm | 0.15 (0.10 to 0.20) | <0.001 | 0.15 (0.10 to 0.19) | <0.001 |
| Breastfeeding | -0.17 (-0.21 to -0.12) | <0.001 | -0.09 (-0.17 to -0.02) | 0.01 |
| Dietary diversity | 0.03 (0.02 to 0.04) | <0.001 | 0.02 (0.006 to 0.03) | 0.003 |
| Mother literate | 0.003 (-0.06 to 0.06) | 0.92 | 0.03 (-0.03 to 0.08) | 0.41 |
| Site  1  2  3  4 | Ref  0.12 (0.06 to 0.18)  0.09 (0.02 to 0.15)  0.03 (-0.04 to 0.09) | <0.001  0.009  0.41 | Ref  0.10 (0.04 to 0.16)  0.09 (0.03 to 0.16)  0.03 (-0.03 to 0.10) | 0.001  0.004  0.31 |
| Admission month | 0.004 (-0.0009 to 0.009) | 0.11 | 0.004 (-0.0008 to 0.009) | 0.10 |

^1^Adjusted for admission weight and time since admission; ^2^Adjusted for admission weight, time since admission, age at admission, sex, amoxicillin treatment arm, breastfeeding status, dietary diversity, mother’s literacy, mother’s age, site, and calendar month.

## **Supplemental Table 2.8.** Model for malaria predicting HAZ

|  | **Univariate** | | **Multivariable^1^** | |
| --- | --- | --- | --- | --- |
|  | *Mean (95% CI)* | *P* | *Adjusted Mean*  *(95% CI)* | *P* |
| Malaria | 0.005 (-0.01 to 0.02) | 0.55 | -0.02 (-0.04 to -0.008) | 0.002 |
| Child’s age | 0.008 (0.007 to 0.009) | <0.001 | 0.007 (0.005 to 0.008) | <0.001 |
| Child’s sex | 0.004 (-0.01 to 0.02) | 0.62 | 0.007 (-0.006 to 0.02) | 0.30 |
| Baseline hemoglobin | -0.007 (-0.01 to -0.004) | <0.001 | -0.005 (-0.008 to -0.002) | 0.003 |
| Amoxicillin treatment arm | 0.007 (-0.008 to 0.02) | 0.37 | 0.006 (-0.007 to 0.02) | 0.40 |
| Breastfeeding | -0.11 (-0.13 to -0.10) | <0.001 | -0.03 (-0.05 to -0.008) | 0.007 |
| Dietary diversity | 0.007 (0.003 to 0.01) | 0.001 | 0.0008 (-0.003 to 0.004) | 0.68 |
| Mother literate | -0.01 (-0.03 to 0.008) | 0.27 | -0.01 (-0.03 to 0.005) | 0.17 |
| Site  1  2  3  4 | Ref  0.02 (0.005 to 0.04)  0.02 (0.004 to 0.05)  -0.04 (-0.06 to -0.02) | 0.01  0.02  <0.001 | Ref  0.03 (0.01 to 0.05)  0.04 (0.02 to 0.06)  -0.03 (-0.05 to -0.01) | 0.001  <0.001  0.001 |
| Admission month | -0.004 (-0.006 to -0.002) | <0.001 | -0.004 (-0.006 to -0.001) | <0.001 |

^1^Adjusted for admission weight and time since admission; ^2^Adjusted for admission weight, time since admission, age at admission, sex, amoxicillin treatment arm, breastfeeding status, dietary diversity, mother’s literacy, mother’s age, site, and calendar month.

## **Supplemental Table 2.9.** Model for malaria predicting WAZ

|  | **Univariate** | | **Multivariable^1^** | |
| --- | --- | --- | --- | --- |
|  | *Mean (95% CI)* | *P* | *Adjusted Mean*  *(95% CI)* | *P* |
| Malaria | 0.05 (0.02 to 0.09) | 0.006 | 0.05 (0.009 to 0.09) | 0.02 |
| Child’s age | 0.009 (0.006 to 0.01) | <0.001 | 0.004 (0.0001 to 0.007) | 0.04 |
| Child’s sex | -0.09 (-0.13 to -0.05) | <0.001 | -0.09 (-0.13 to -0.06) | <0.001 |
| Baseline hemoglobin | -0.003 (-0.01 to 0.005) | 0.49 | 0.007 (-0.002 to 0.02) | 0.15 |
| Amoxicillin treatment arm | 0.11 (0.08 to 0.15) | <0.001 | 0.11 (0.07 to 0.14) | <0.001 |
| Breastfeeding | -0.16 (-0.20 to -0.12) | <0.001 | -0.11 (-0.17 to -0.05) | <0.001 |
| Dietary diversity | 0.02 (0.01 to 0.03) | <0.001 | 0.01 (0.005 to 0.02) | 0.003 |
| Mother literate | 0.003 (-0.04 to 0.05) | 0.89 | 0.005 (-0.04 to 0.05) | 0.84 |
| Site  1  2  3  4 | Ref  0.07 (0.02 to 0.12)  0.01 (-0.04 to 0.06) -0.05 (-0.10 to -0.002) | 0.005  0.70  0.04 | Ref  0.06 (0.008 to 0.10)  0.02 (-0.03 to 0.07)  -0.03 (-0.08 to 0.02) | 0.02  0.45  0.24 |
| Admission month | 0.0009 (-0.003 to 0.004) | 0.59 | 0.002 (-0.002 to 0.005) | 0.30 |

^1^Adjusted for admission weight and time since admission; ^2^Adjusted for admission weight, time since admission, age at admission, sex, amoxicillin treatment arm, breastfeeding status, dietary diversity, mother’s literacy, mother’s age, site, and calendar month.

## **Supplemental Table 2.10.** Model for malaria predicting MUAC

|  | **Univariate** | | **Multivariable^1^** | |
| --- | --- | --- | --- | --- |
|  | *Mean (95% CI)* | *P* | *Adjusted Mean*  *(95% CI)* | *P* |
| Malaria | 0.05 (0.01 to 0.09) | 0.008 | 0.03 (-0.01 to 0.07) | 0.20 |
| Child’s age | 0.01 (0.009 to 0.1) | <0.001 | 0.01 (0.008 to 0.02) | <0.001 |
| Child’s sex | -0.13 (-0.17 to -0.09) | <0.001 | -0.13 (-0.17 to -0.09) | <0.001 |
| Baseline hemoglobin | -0.01 (-0.02 to -0.006) | 0.001 | -0.002 (-0.01 to 0.007) | 0.65 |
| Amoxicillin treatment arm | 0.10 (0.07 to 0.14) | <0.001 | 0.10 (0.07 to 0.14) | <0.001 |
| Breastfeeding | -0.14 (-0.18 to -0.10) | <0.001 | 0.02 (-0.04 to 0.08) | 0.51 |
| Dietary diversity | 0.01 (0.005 to 0.03) | 0.004 | 0.01 (0.003 to 0.02) | 0.01 |
| Mother literate | -0.01 (-0.06 to 0.04) | 0.62 | -0.005 (-0.05 to 0.04) | 0.83 |
| Site  1  2  3  4 | Ref  -0.07 (-0.12 to -0.02)  0.05 (0.002 to 0.11)  -0.001 (-0.05 to 0.05) | 0.007  0.04  0.96 | Ref  -0.07 (-0.12 to -0.02)  0.06 (0.01 to 0.11)  -0.002 (-0.05 to 0.05) | 0.003  0.01  0.94 |
| Admission month | -0.002 (-0.006 to 0.002) | 0.28 | -0.002 (-0.006 to 0.001) | 0.21 |

^1^Adjusted for admission weight and time since admission; ^2^Adjusted for admission weight, time since admission, age at admission, sex, amoxicillin treatment arm, breastfeeding status, dietary diversity, mother’s literacy, mother’s age, site, and calendar month.
