# Supplementary material for: Malaria and Nutritional Status Among Children With Severe Acute Malnutrition in Niger: A Prospective Cohort Study
Source: Clin Infect Dis. 2018 Mar 7;67(7):1027–34. doi: 10.1093/cid/ciy207 (PMC6137121; doi:10.1093/cid/ciy207)
Supplement: Supplementary Tables [file ciy207_suppl_supplemental_tables.docx]

**Oldenburg et al**

**SUPPLEMENTAL MATERIAL**

**Supplemental Table 1.** *Association between time-updated anthropometric indices and baseline dietary indicators with the risk of first malaria infection during SAM treatment among children admitted during the malaria season (N=581)*

|  | **Unadjusted** | | **Adjusted^1^** | |
| --- | --- | --- | --- | --- |
|  | *HR (95% CI)* | *P-trend* | *aHR (95% CI)^1^* | *P-trend* |
| WHZ  ≥-2  <-2 and ≥-3  < -3 | 1.00  1.09 (0.82 to 1.46)  0.84 (0.53 to 1.33) | 0.55 | 1.00  1.02 (0.76 to 1.37)  0.75 (0.47 to 1.19) | 0.37 |
| HAZ  ≥-2  <-2 and ≥-3  < -3 | 1.00  1.26 (0.85 to 1.86)  0.94 (0.65 to 1.35) | 0.13 | 1.00  1.31 (0.88 to 1.95)  0.99 (0.68 to 1.45) | 0.57 |
| WAZ  ≥-2  <-2 and ≥-3  < -3 | 1.00  0.85 (0.54 to 1.34)  0.83 (0.54 to 1.28) | 0.69 | 1.00  0.85 (0.54 to 1.34)  0.87 (0.55 to 1.36) | 0.71 |
| MUAC <115 | 0.80 (0.58 to 1.10) | 0.17 | 0.77 (0.55 to 1.07) | 0.12 |
| Household food insecurity index | 1.01 (0.99 to 1.02) | 0.45 | 1.01 (0.99 to 1.02) | 0.53 |
| Dietary diversity score | 1.04 (0.96 to 1.12) | 0.39 | 1.00 (0.92 to 1.09) | 0.95 |
| Current breastfeeding | 0.80 (0.62 to 1.04) | 0.10 | 0.74 (0.49 to 1.11) | 0.14 |
| Hemoglobin, g/dL | 1.05 (0.97 to 1.14) | 0.26 | 1.00 (0.92 to 1.10) | 0.92 |

^1^Adjusted for including child’s age and sex, mother’s literacy, number of children in the household under the age of 5, household bednet use, breastfeeding status, study site, calendar month, and cough, vomiting, diarrhea at admission.

**Supplemental Table 2:** *Association between malaria at admission with nutritional recovery (n = 1,344) and response to treatment among children who recovered from SAM treatment (n=837) among children admitted during the malaria season*

|  | **Univariate** | | **Multivariable** | |
| --- | --- | --- | --- | --- |
|  | HR | *P* | aHR | *P* |
| Nutritional recovery | 1.20 (0.92 to 1.56) | 0.20 | 1.00 (0.84 to 1.20) | 0.96 |
|  | *Mean (95% CI)^1^* |  | *Adjusted Mean*  *(95% CI)^2^* |  |
| Time until recovery (days) | -1.15  (-2.53 to 0.24) | 0.10 | -0.57 (-2.28 to 1.14) | 0.52 |
| Mean weight change (kg) | 0.04  (-0.009 to 0.08) | 0.12 | 0.02  (-0.03 to 0.07) | 0.45 |
| Weight gain (g/kg/day) | 0.27  (-0.13 to 0.67) | 0.18 | 0.31  (-0.14 to -0.77) | 0.18 |
| Mean height change (cm) | -0.06  (-0.10 to -0.01) | 0.01 | -0.07  (-0.12 to -0.02) | 0.01 |
| Height change (mm/day) | -0.002  (-0.004 to -0.0005) | 0.01 | -0.003  (-0.005 to -0.0004) | 0.02 |
| WHZ | 0.09  (0.03 to 0.16) | 0.003 | 0.06  (-0.02 to 0.13) | 0.13 |
| HAZ | -0.007  (-0.03 to 0.01) | 0.49 | -0.03 (-0.05 to -0.006) | 0.01 |
| WAZ | 0.06  (0.007 to 0.11) | 0.03 | 0.03  (-0.03 to 0.08) | 0.36 |
| MUAC | 0.01  (-0.04 to 0.06) | 0.62 | -0.03  (-0.09 to 0.03) | 0.28 |

^1^Adjusted for admission weight and time since admission; ^2^Adjusted for admission weight, time since admission, age at admission, sex, amoxicillin treatment arm, breastfeeding status, dietary diversity, mother’s literacy, mother’s age, site, and calendar month.

**Supplemental Table 3:** *Association between number of malaria episodes over the follow-up period with nutritional recovery (n = 2399) and response to treatment among children who recovered from SAM treatment (n=1542). Effect sizes should be interpreted as effect per one additional malaria episode over the follow-up period.*

| **Outcome** | **Univariate** | | **Multivariable** | |
| --- | --- | --- | --- | --- |
|  | HR | *P* | aHR | *P* |
| Nutritional recovery | 1.05 (0.99 to 1.12) | 0.13 | 0.97 (0.91 to 1.04) | 0.40 |
|  | *Mean (95% CI)^1^* |  | *Adjusted Mean*  *(95% CI)^2^* |  |
| Time until recovery (days) | -0.15 (-0.85 to 0.56) | 0.68 | 0.23 (-0.50 to 0.96) | 0.54 |
| Mean weight change (kg) | -0.01 (-0.03 to 0.01) | 0.35 | -0.01 (-0.03 to 0.01) | 0.31 |
| Weight gain (g/kg/day) | -0.04 (-0.24 to 0.15) | 0.67 | -0.01 (-0.20 to 0.18) | 0.92 |
| Mean height change (cm) | -0.02 (-0.05 to 0.0007) | 0.06 | -0.02 (-0.05 to 0.002) | 0.08 |
| Height change (mm/day) | -0.001 (-0.002 to 0.00005) | 0.06 | -0.0009 (-0.002 to 0.0002) | 0.11 |
| WHZ | 0.02 (-0.02 to 0.05) | 0.34 | -0.0003 (-0.03 to 0.03) | 0.87 |
| HAZ | 0.0005 (-0.009 to 0.01) | 0.92 | -0.009 (-0.02 to 0.00001) | 0.05 |
| WAZ | 0.003 (-0.02 to 0.03) | 0.84 | -0.01 (-0.04 to 0.01) | 0.41 |
| MUAC | 0.0008 (-0.02 to 0.03) | 0.95 | -0.02 (-0.04 to 0.008) | 0.17 |

^1^Adjusted for admission weight and time since admission; ^2^Adjusted for admission weight, time since admission, age at admission, sex, amoxicillin treatment arm, breastfeeding status, dietary diversity, mother’s literacy, mother’s age, site, and calendar month.
